# Risk factors and racial disparities related to low maternal birth satisfaction with labor induction: a prospective, cohort study

**DOI:** 10.1186/s12884-019-2658-z

**Published:** 2019-12-30

**Authors:** Rebecca F. Hamm, Sindhu K. Srinivas, Lisa D. Levine

**Affiliations:** Department of Obstetrics & Gynecology, Maternal and Child Health Research Center, University of Pennsylvania Perelman School of Medicine, 3400 Spruce Street, 2 Silverstein, Philadelphia, PA 19104 USA

**Keywords:** Satisfaction, Labor induction, Racial disparities, Race

## Abstract

**Background:**

Decreased birth satisfaction has been associated with labor induction. Yet, there is a paucity of data evaluating risk factors for decreased satisfaction associated with labor induction. We aimed to determine what factors impact low birth satisfaction in labor induction and evaluate racial disparities in birth satisfaction.

**Methods:**

We performed a prospective cohort study of women with term, singleton gestations undergoing labor induction at our institution from Jan 2018 to Jun 2018. Women completed the validated Birth Satisfaction Scale-Revised postpartum, which is subdivided into 3 domains: (1) quality of care provision, (2) women’s personal attributes, and (3) stress experienced during labor. A total satisfaction score above the mean was classified as “satisfied”, and below as “unsatisfied.” Domain and item scores were compared by race.

**Results:**

Three hundred thirty of 414 (79.7%) eligible women were included. There was no significant difference in birth satisfaction by age, body mass index, Bishop score, or labor induction agent. Black women were 75% more likely to be unsatisfied than non-Black women (54.0% vs. 37.2%, OR 1.75 [95% CI 1.11–2.76], *p* = 0.037), nulliparas were 71% more  likely to be unsatisfied than multiparas (54.2% vs. 40.9%, OR 1.71 [95% CI 1.09–2.67], *p* = 0.019), and women whose labor resulted in cesarean birth were almost 3 times more likely to be unsatisfied than women with a vaginal birth (67.4% vs. 42.3%, OR 2.82 [95% CI 1.69–4.70], *p* < 0.001). Additionally, increased labor length quartile was associated with decreased satisfaction >(*p* = 0.003). By race, domain 3 scores, which reflect preparedness for labor, were lower for Black women. No differences were seen for domain 1 or 2.

**Conclusions:**

Black race, cesarean birth, and increasing labor length were identified as risk factors for low birth satisfaction among women who underwent labor induction. Further studies should explore interventions to target women at risk for low birth satisfaction.

## Background

The experience of the birth process can be of momentous importance in a woman’s life. Maternal satisfaction with birth also has notable downstream effects on maternal and neonatal health. In prior studies, maternal satisfaction with the labor process has been directly linked to healthy mother/baby bonding, improved breastfeeding rates, and a decreased risk for postpartum depression [1, 2]. Prior work examining maternal birth satisfaction has demonstrated its association with induction of labor (IOL) [3, 4]. Shetty et al. compared the birth experience for 450 women undergoing induction of labor with 450 women in spontaneous labor. Significantly more women were satisfied with their labor in the spontaneous labor group than the labor induction group (79.5% vs. 70.4%, *p* = 0.006). Specifically, a longer labor induction length appeared to play a significant role, with the labor induction agent and more vaginal examinations perceived as secondary issues [3].

While labor induction is a known risk factor for low maternal birth satisfaction, there is a paucity of data evaluating risk factors for low maternal satisfaction associated specifically with IOL. This is a critical population to focus on, since we know IOL is one of the most common obstetrical procedures. In the United States alone, over four million women give birth annually, with more than 20% of them undergoing an IOL. This equates to more than 1 million pregnant women in the US undergoing a labor induction per year [5].

Thus, our primary aim was to determine what factors impact maternal birth satisfaction in women undergoing IOL. The ultimate goal of understanding these risk factors would be to target those women at highest risk for low maternal satisfaction both before starting, as well as throughout, the labor induction process.

Secondarily, we aimed to determine if maternal birth satisfaction in women undergoing IOL differed by race. Racial disparities are present in most obstetric outcomes, with Black women at greater risk of perinatal morbidity than non-Black women [6, 7]. Racial disparities have been seen in satisfaction with other aspects of reproductive health, such as contraceptive services and prenatal care [8, 9]. If racial disparities exist in maternal birth satisfaction related to IOL, this could provide a key target for interventions to improve not only the outcome of maternal satisfaction, but also the downstream perinatal outcomes.

## Methods

We performed a prospective cohort study on women undergoing IOL at one tertiary care institution from January 2018 to June 2018. This study was performed concurrently with another study at our institution regarding IOL, and thus inclusion and exclusion criteria of that parent study were utilized here. Institutional Review Board approval and a Waiver of Documentation of Informed Consent was obtained from our institution. Survey completion was considered consent. All English-speaking women with term (≥37 weeks) singleton gestations undergoing IOL with an unfavorable cervix (defined by our group as ≤2 cm dilation and Bishop score ≤ 6) and intact membranes delivering at our institution during the study period were approached for survey completion and were included. Both nulliparous and multiparous women were included. Women with a prior cesarean section, HIV, medical conditions requiring an assisted second stage, HELLP (Hemolysis, Elevated Liver Enzymes, and Low Platelets) syndrome or eclampsia, and intrauterine growth restriction with abnormal Doppler studies were excluded. Labor induction at our institution is currently managed at the discretion of the provider, with variation by provider and by patient in terms of labor induction agent, frequency of cervical examinations, and timing of amniotomy. At our institution, misoprostol is given as a 25mcg dose vaginally, and can be given as frequently as every 3 h. Misoprostol can also be used concurrently with an intracervical balloon catheter. Our oxytocin protocol begins with 2 milliunits/minute of oxytocin increasing by 2 milliunits every 15 min until regular uterine contractions occur. Forty milliunits of oxytocin is considered the maximum dose; however, there is no limit as to the length of time a participant can remain at 40 milliunits.

On the first postpartum day, eligible women were approached while admitted to the postpartum unit by a trained research assistant. If the patient agreed to participate, she was offered to self-complete a modified version of the validated 10 question Birth Satisfaction Scale-Revised (BSS-R). As a Waiver of Documentation of Informed Consent was obtained for this study, survey completion was considered as consent. The BSS-R has been demonstrated to be a robust, valid, and reliable multidimensional psychometric instrument for measuring postnatal women’s birth satisfaction in diverse populations [10, 11]. The BSS-R asks women to report agreement or disagreement with 10 statements using a 5-point Likert scale. The total BSS-R ranges from 10 to 50 with higher scores indicating increased satisfaction. The BSS-R is subdivided into 3 domains of satisfaction: (1) quality of care provision, (2) women’s personal attributes, and (3) stress experienced during labor.

Our primary aim was to determine what factors impact birth satisfaction in women undergoing IOL. Previous literature utilizing the BSS-R does not delineate a clear cutoff for determining satisfaction. Thus, for our primary analysis, women with a BSS-R score above the mean were classified as “satisfied” and women with a BSS-R below the mean were classified as “unsatisfied”. Our secondary aim was to determine if there are racial disparities in maternal birth satisfaction for women undergoing IOL. Thus, for our secondary analysis, women were grouped by self-reported race elicited during their initial prenatal visit as Black or non-Black. Total, domain, and individual item scores were compared by race.

Descriptive statistics for continuous variables are presented as mean and standard deviation or median and interquartile range, where appropriate. Categorical variables are presented as frequencies and percentages. Univariate analysis was performed using Chi-square for categorical and Wilcoxon rank sum for continuous variables. Multivariable analysis was performed using logistic regression. Demographic and clinical characteristics there were associated on bivariate tests (*p* < 0.05) with the grouping variable and the outcome of interest were evaluated as potential covariates for regression models. Backwards stepwise elimination of covariates (with *p*-value > 0.10 for removal) was performed for each regression model to determine which covariates would be retained in the final model. Statistical analyses were performed with STATA version 15 (STATA Corporation, College Station, TX). Statistical significance was set at *p* < 0.05.

## Results

Our institution performs approximately 4200 deliveries per year with more than 20% of them undergoing labor induction. During the study period, there were 414 women who underwent an IOL with an unfavorable cervix and met criteria for our current study. Of these, 330 (79.7%) women completed the BSS-R and were included in the analysis. Approximately two-thirds (64.6%, *n* = 213) of our population was Black. Of included women, 203 (61.5%) were nulliparous. The top 3 indications for labor induction were maternal indications (39.7%), fetal heart rate indications (12.1%), and elective (11.2%). Examples of maternal indications include: chronic hypertension, gestational hypertension, preeclampsia, diabetes, renal disease, history of venous thromboembolism, and cardiac disease or other chronic medical condition where labor induction was recommended. Examples of fetal indications include: oligohydramnios, intrauterine growth restriction, and abnormality on fetal testing. Full baseline demographic data is described in Table 1.

**Table 1:** Baseline demographics of study sample: women undergoing labor induction

Age ^a^	29 [25–34]
Race	
*Black*	213 (64.6)
*Non-Black*	117 (35.4)
Ethnicity	
*Non-Hispanic*	316 (95.8)
*Hispanic*	14 (4.2)
Insurance Type	
*Private/Individual*	152 (46.1)
*Medicaid/Uninsured*	178 (53.9)
BMI at Delivery	
*< 25*	24 (7.3)
*25–29.9*	84 (25.5)
*30–34.9*	103 (31.2)
*35–39.9*	53 (16.1)
*≥ 40*	66 (20.0)
Number of prenatal visits ^a^	11 [8–13]
Parity	
*Nulliparous*	203 (61.5)
*Multiparous*	127 (38.5)
Pregestational diabetes	8 (2.4)
Chronic hypertension	23 (7.0)
Indication for labor induction	
*Maternal indications* ^*b*^	131 (39.7)
*Fetal indications* ^*c*^	114 (34.6)
*Elective/ Other* ^*d*^	49 (14.9)
*Late term*	36 (10.9)
Gestational Age at Delivery (weeks) ^a^	39 [38–40]
Modified Bishop Score at start of labor induction ^a^	2 [1–3]
Cervical dilation at start of labor induction	2 [0–2]
Labor induction agent	
*Misoprostol alone*	11 (3.3)
*Foley alone*	15 (4.6)
*Foley + Misoprostol*	265 (80.3
*Foley + Pitocin*	37 (11.2)
*Other*	2 (0.6)
Regional Anesthesia	312 (94.6)

Data are presented as n(%) unless otherwise indicated

^a^Median [Interquartile Range]

^b^Examples include: chronic hypertension, gestational hypertension, preeclampsia, diabetes, renal disease, history of venous thromboembolism, cardiac disease or other chronic medical condition where labor induction was recommended

^c^Examples include: Oligohydramnios, intrauterine growth restriction, abnormality on fetal testing

^d^Examples of “other” include: history of an intrauterine fetal demise, vaginal bleeding at term, cholestasis

The mean BSS-R score among all women was 38.2 out of a possible 50 (± 5.5). For the primary analysis, women were determined to be “satisfied” if total BSS-R score was > 38 (*n* = 168; 50.9%) and “unsatisfied” if total BSS-R score was ≤38 (*n* = 162; 49.1%). Table 2 shows the percentage of “unsatisfied” women for each risk factor. Self-identified Black women were 75% more likely to be unsatisfied than non-Black women (54.0% vs. 37.2%, OR 1.75 [95%CI 1.11–2.76], *p* = 0.037), nulliparas were 71% more likely to be unsatisfied than multiparas (54.2% vs. 40.9%, OR 1.71 [95%CI 1.09–2.67], *p* = 0.019), and women whose labor resulted in cesarean birth were almost 3 times more likely to be unsatisfied than women with a vaginal birth (67.4% vs. 42.3%, OR 2.82 [95%CI 1.69–4.70], *p* < 0.001) (Table 2). Bishop score and cervical dilation at the start of labor induction did not have an impact on maternal satisfaction.

**Table 2:** Risk factors for decreased birth satisfaction among women undergoing labor induction

	Unsatisfied (*n* = 162)	Satisfied (*n* = 168)	*p*-value
Age *a*	29 [24–34]	29 [25–34]	0.53
Race			0.02
*Black (n = 213)*	115 (54.0)	98 (46.0)	
*Non-Black (n = 117)*	47 (40.2)	70 (59.8)	
Insurance Type			0.31
*Private/Individual (n = 152)*	70 (46.1)	82 (54.0)	
*Medicaid/Uninsured (n = 178)*	92 (51.7)	86 (48.3)	
BMI			0.99
*< 25 (n = 24)*	11 (45.8)	13 (54.2)	
*25–29.9 (n = 84)*	40 (47.6)	44 (52.4)	
*30–34.9 (n = 103)*	51 (49.5)	52 (50.5)	
*35–39.9 (n = 53)*	26 (49.1)	27 (50.9)	
*> or equal to 40 (n = 66)*	34 (51.5)	32 (48.5)	
Number of Prenatal Visits ^a^	10 [8–13]	11 [9–13]	0.21
Parity			0.02
*Nulliparous (n = 203)*	110 (54.2)	93 (45.8)	
*Multiparous (n = 127)*	52 (40.9)	75 (59.1)	
Indication for labor induction			0.31
*Maternal indications (n = 131)* ^b^	58 (44.3)	73 (55.7)	
*Fetal indications (n = 114)* ^c^	56 (49.1)	58 (50.9)	
*Elective/Other (n = 49)* ^d^	26 (53.1)	23 (46.9)	
*Late term (n = 36)*	22 (61.1)	14 (38.9)	
Gestational Age at Delivery (weeks) ^a^	39 [38–40]	39 [38–40]	0.09
Modified Bishop Score at labor induction ^a^	3 [1–3]	2 [2–3]	0.84
Cervical dilation at labor induction ^a^	1–2 [0–2]	2 [0–2]	0.41
Labor induction Method			0.48
*Misoprostol alone (n = 11)*	5 (45.5)	6 (54.6)	
*Foley alone (n = 15)*	9 (60.0)	6 (40.0)	
*Foley + Misoprostol (n = 265)*	126 (47.6)	139 (52.5)	
*Foley + Pitocin (n = 37)*	20 (54.1)	17 (46.0)	
*Other (n = 2)*	2 (100)	0 (0)	
Mode of Delivery			< 0.0001
*Vaginal Delivery (n = 241)*	102 (42.3)	139 (57.7)	
*Cesarean Delivery (n = 89)*	60 (67.4)	29 (32.6)	
Length of labor (h)^a^	18.3 [12.5–23.1]	14.6 [10.6–21.2]	0.0013
Indication for Cesarean			0.65
*Elective/Other (n = 7)*	5 (71.4)	2 (28.6)	
*Failed labor induction/Arrest of active phase (n = 37)*	26 (70.3)	11 (29.7)	
*Arrest of descent/Failed operative delivery (n = 13)*	10 (77.0)	3 (23.1)	
*Fetal heart rate indications (n = 32)*	19 (59.4)	13 (40.6)	
Regional Anesthesia			0.17
*Yes (n = 312)*	156 (50.0)	156 (50.0)	
*No (n = 18)*	6 (33.3)	12 (66.7)	

Data are presented as the row n(%) unless otherwise indicated

^a^Row Median [Interquartile Range]

^b^Examples include: chronic hypertension, gestational hypertension, preeclampsia, diabetes, renal disease, history of venous thromboembolism, cardiac disease or other chronic medical condition where labor induction was recommended

^c^Examples include: Oligohydramnios, intrauterine growth restriction, abnormality on fetal testing

^d^Examples of “other” include: history of an intrauterine fetal demise, vaginal bleeding at term, cholestasis

**Fig. 1:**
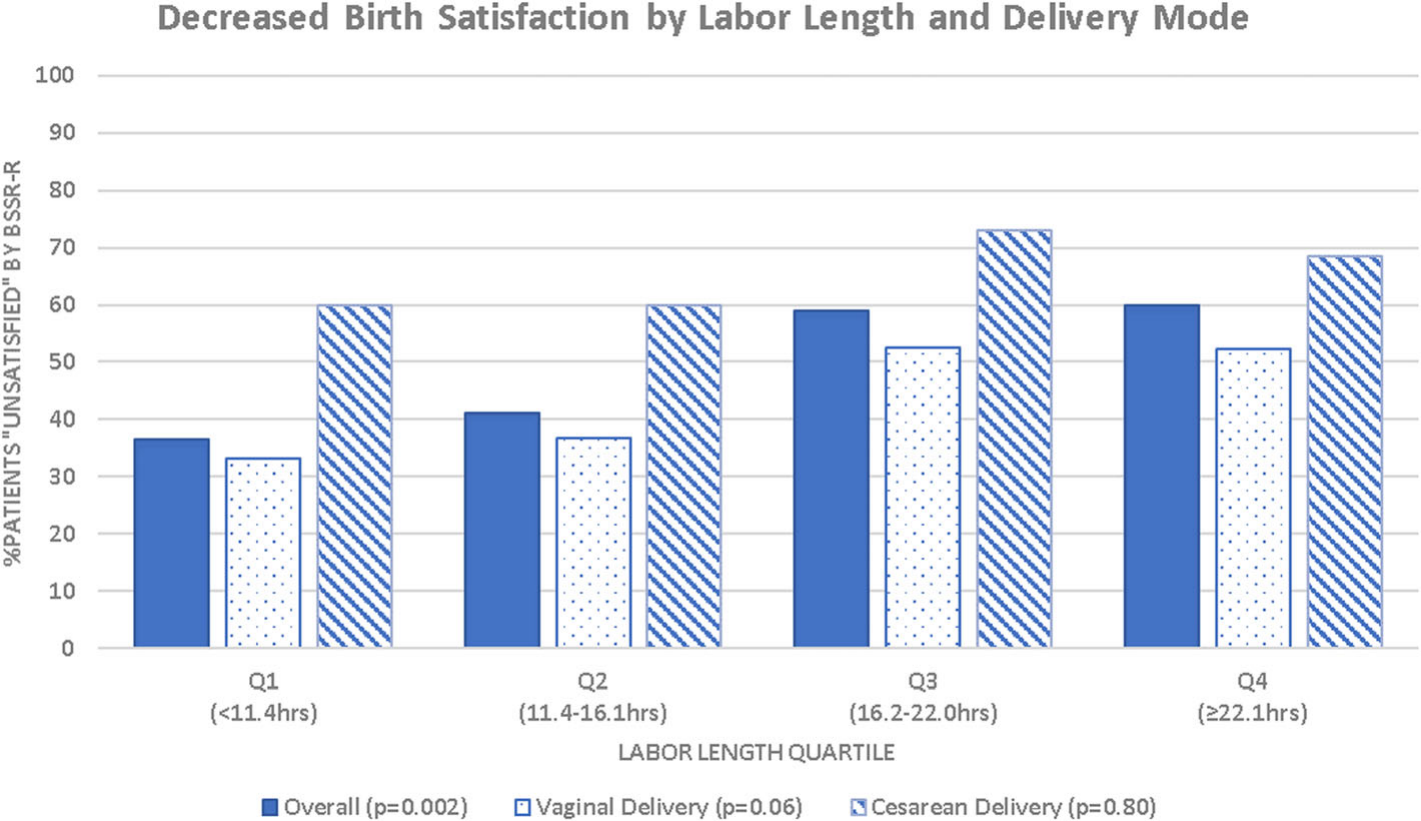
Decreased birth satisfaction by labor length and delivery mode

Overall, median labor length, calculated as the time from placement of first labor induction agent to time of delivery, was 16.2 [11.4–22.1] hours. Increasing labor length quartile was associated with decreased maternal birth satisfaction (*p* = 0.002). This association remained even when restricting the analysis to women that achieved a vaginal birth (Fig. 1). In multivariable analysis, nulliparity was no longer an independent predictor of maternal birth satisfaction, but race, mode of delivery, and labor length quartile remained independent risk factors for low maternal birth satisfaction (Table 3).

**Table 3:** Multivariate analysis of factors associated with low maternal birth satisfaction during labor induction

Risk Factor		aOR	95%CI	*p*-value
Race	*Black*	REF	–	–
	*Non-Black*	1.78	1.10–2.90	0.02
Mode of Delivery	*Vaginal*	REF	–	–
	*Cesarean*	0.46	0.27–0.79	0.005
Labor Length Quartile	*1 (< 11.4 h)*	REF	–	–
	*2 (11.4–16.1 h)*	0.77	0.40–1.48	0.44
	*3 (16.2–22.0 h)*	0.40	0.20–0.76	0.006
	*4 (≥22.1 h)*	0.47	0.24–0.91	0.03

REF = reference group

For our secondary analysis, women were grouped as Black (*n* = 213, 64.6%) or non-Black (*n* = 117, 35.4%). Black women were younger (26 [23–31] vs. 32 [30–36] years old, *p* < 0.0001), more likely to have Medicaid or be uninsured (77% vs. 12%, *p* < 0.0001), and more likely to be multiparous (46.5% vs. 23.9%, *p* < 0.0001) (Table 4). Black women had lower median starting modified Bishop scores (2 [2, 3] vs. 3 [1–4], *p* = 0.04). Finally, Black women were significantly more likely to undergo cesarean section in this cohort than non-Black women (31.5% vs. 18.8%, *p* = 0.013).

**Table 4:** Demographic and clinical outcomes of study group of women undergoing labor induction by race

	Black (*n* = 213)	Non-Black (*n* = 117)	*p*-value
Age ^a^	26 [23–31]	32 [30–36]	< 0.0001
Insurance Type			< 0.0001
*Private/Individual*	49 (23.0)	103 (88.0)	
*Medicaid/Uninsured*	164 (77.0)	14 (12.0)	
BMI			0.001
*< 25*	13 (6.1)	11 (9.4)	
*25–29.9*	42 (19.7)	42 (35.9)	
*30–34.9*	65 (30.5)	38 (32.5)	
*35–39.9*	40 (18.8)	13 (11.1)	
*> or equal to 40*	53 (24.9)	13 (11.1)	
Number of Prenatal Visits ^a^	10 (7–12)	12 (10–13)	< 0.0001
Parity			< 0.0001
*Nulliparous*	114 (53.5)	89 (76.1)	
*Multiparous*	99 (46.5)	28 (23.9)	
Indication for labor induction			0.012
*Maternal* ^b^	83 (39.0)	48 (41.0)	
*Fetal* ^c^	85 (39.9)	29 (24.8)	
*Elective/Other* ^d^	28 (13.2)	21 (18.0)	
*Late term*	17 (8.0)	19 (16.2)	
Gestational Age at Delivery (weeks) ^a^	39 [38–40]	39 [38–40]	0.06
Scheduled IOL	90 (42.3)	70 (59.8)	0.002
Modified Bishop Score at labor induction ^a^	2 [2–3]	3 [1–4]	0.04
Cervical dilation at labor induction ^a^	1–2 [0–2]	1–2 [0–2]	0.86
Labor induction Method			0.25
*Misoprostol alone*	8 (3.8)	3 (2.6)	
*Foley alone*	10 (4.7)	5 (4.3)	
*Foley + Misoprostol*	164 (77.0)	101 (86.3)	
*Foley + Pitocin*	29 (13.6)	8 (6.8)	
*Other*	2 (0.9)	0 (0.0)	
Mode of Delivery			0.013
*Vaginal Delivery*	146 (68.5)	95 (81.2)	
*Cesarean Delivery*	67 (31.5)	22 (18.8)	
Length of labor (hrs) ^a^	15.9 [10.6–22.3]	16.4 [12.4–21.7]	0.29
Labor Length Quartile			0.009
*1(< 11.4 h)*	63 (29.6)	19 (16.2)	
*2 (11.4–16.1 h)*	47 (22.1)	36 (30.8)	
*3 (16.2–22.0 h)*	46 (21.6)	37 (31.6)	
*4(≥22.1 h)*	57 (26.8)	25 (21.4)	
Indication for Cesarean			< 0.0001
*Elective/Other*	5 (7.5)	2 (9.1)	
*Failed labor induction/Arrest of active phase*	33 (49.3)	4 (18.2)	
*Arrest of descent/Failed operative delivery*	3 (4.5)	10 (45.5)	
*Fetal heart rate indications*	26 (38.8)	6 (27.3)	
Regional Anesthesia	200 (93.9)	112 (95.7)	0.48

^a^Median [Interquartile Range]

^b^Examples include: chronic hypertension, gestational hypertension, preeclampsia, diabetes, renal disease, history of venous thromboembolism, cardiac disease or other chronic medical condition where labor induction was recommended

^c^Examples include: Oligohydramnios, intrauterine growth restriction, abnormality on fetal testing

^d^Examples of “other” include: history of an intrauterine fetal demise, vaginal bleeding at term, cholestasis

Black women had lower median total BSS-R score than non-Black women (38 [36–43] vs. 40 [34–42], *p* = 0.02). While there was no difference in domain 1 (stress experienced during labor), there were significant differences between Black and non-Black women for the individual questions in that domain (Table 5). Specifically, Black women were more likely to state that their “labor was excessively long”; even when achieving a vaginal birth and despite the fact that actual labor length did not differ by race (Table 4). In contrast, Black women were more likely to report agreement with the statements “I came through labor unharmed” and “I was not distressed at all during labor”. There were no differences between Black and non-Black women for domain 2 (quality of care provision). Scores for domain 3 (women’s personal attributes), which reflects underlying anxiety and preparedness for labor, were lower for Black women than non-Black women.

**Table 5:** Birth Satisfaction Scale-Revised scores for women undergoing labor induction by race

	Black	Non-Black	*p*-value
Total BSS-R (out of 50) ^a^	38 [34–42]	40 [36–43]	0.02
Domain #1: Stress Experienced During Labor (out of 20) ^a^	14 [12–16]	14 [12–16]	0.40
*“I came through childbirth virtually unharmed.”*	90.1	80.3	0.01
*“I thought my labor was excessively long.”*	34.3	21.4	0.01
*“I found giving birth a distressing experience.”*	31.5	26.5	0.35
*“I was not distressed at all during labor.”*	35.2	21.4	0.01
Domain #2: Quality of Care Provision (out of 20) ^a^	18 [16–20]	19 [17–20]	0.06
*“The delivery room staff encouraged me to make decisions about how I wanted my birth to progress.”*	83.6	88.0	0.28
*“I felt well supported by staff during my labor and delivery.”*	94.4	97.4	0.20
*“The staff communicated well with me during labor.”*	96.7	95.7	0.65
*“I was satisfied with how I delivered.”*	85.5	88.9	0.38
Domain #3: Women’s personal attributes (out of 10) ^a^	6 (5–8)	7 (6–9)	0.01
*“I felt very anxious during my labor and delivery.”*	58.2	39.3	0.001
*“I felt out of control during my birth experience.”*	16.4	15.4	0.80

Results shown are the percentage of women who agreed or strongly agreed with the statement, unless otherwise stated

^a^Median [Interquartile Range]

## Discussion

In this observational cohort, we demonstrated that Black race, cesarean birth, and increasing labor length were independent risk factors for low birth satisfaction among women who underwent labor induction. Furthermore, we identified racial disparities in birth satisfaction for women undergoing IOL. Specifically, Black women had lower satisfaction with the overall birth process, as well as the domain that reflects preparedness for labor.

Maternal birth satisfaction is important to women and impacts maternal and neonatal morbidity. There is a paucity of data around birth satisfaction for women undergoing labor induction. Prior studies compared women who underwent labor induction with those in spontaneous labor, demonstrating decreased birth satisfaction among women undergoing labor induction and evaluating for underlying causes of that difference [3, 4]. Henderson (2015) performed a mixed methods study, surveying 5333 women who gave birth in the United Kingdom in 2009, 20% of which were induced. In the qualitative analysis, the main thematic concerns that emerged regarding IOL were delay, staff shortages, neglect, as well as pain and anxiety in relation to getting the labor induction started. Shetty (2005) compared 450 women undergoing IOL with 450 women in spontaneous labor, again demonstrating lower maternal birth satisfaction in the IOL group (70.4% vs 79.5%, *p* = 0.006). Of note, each of these studies used differing scales from ours to determine level of maternal birth satisfaction.

While labor inductions may be associated with low maternal satisfaction, many obstetric scenarios necessitate labor induction. In addition, in context of the ARRIVE trial, a large multicenter study performed through the Maternal Fetal Medicine Units (MFMU) Network demonstrating decreased cesarean birth rates as well as lower rates of hypertensive disorders of pregnancy when comparing elective labor induction at 39 weeks vs. expectant management for low risk pregnancies, women may elect for IOL at increasing rates [12]. Interestingly, in that study, women in the labor induction arm reported higher labor agentry scores, indicating an increased sense of control over the labor process, when compared with the spontaneous labor group. Of note, all women in that group elected to participate in the trial knowing there was at least a 50% chance of undergoing a labor induction in the 39th week. Thus, it becomes critical to determine risk factors for low birth satisfaction among women undergoing IOL in order to target these women during the labor induction process.

Prior work examining risk factors for decreased birth satisfaction during labor induction has focused on mode of delivery. Ezeanochie et al. found that among women undergoing IOL in a Nigerian population (*n* = 252), those who delivered via cesarean were significantly more likely to be dissatisfied than those who birthed vaginally (13.3% vs 61.1%, *p* = 0.001) ( [13]). Simpson et al. (*n* = 551) found that more women who had a cesarean reported that they would not want to have an IOL again in comparison to those who had a vaginal delivery (57.4% vs 34%) ( [14]).

Our findings confirm cesarean birth as a risk factor for low maternal birth satisfaction, underscoring that birth mode clearly plays a role in a woman’s overall perception of the birth process. Our data also found increasing labor length as a risk for low maternal birth satisfaction. This highlights women’s appreciation of a faster labor induction time. Our final independent risk factor for low maternal birth satisfaction, Black race, has not previously been demonstrated and required additional probing. When determining which aspects of the survey were most influenced by race, the domain reflecting preparedness for labor most explained this difference. Thus, a gap in prenatal care education or counseling at admission for IOL regarding what to expect in the labor and birth process may explain this disparity. Of note, a difference in mode of birth was also seen by race, a finding that has been observed in other studies [15]. In exploratory analyses, this finding held true when adjusting for confounders including insurance type, parity, and bishop score at start of labor induction. This observation deserves further investigation, as reducing this disparity could improve both maternal satisfaction and maternal morbidity. In addition, in this study, no difference was seen regarding maternal birth satisfaction by Bishop score and cervical dilation at labor induction start. This is likely secondary to our source population, which required an unfavorable cervix for inclusion. Larger differences might have been seen with more heterogeneous starting cervical exams.

This study has significant strengths. With a large percentage of Black women, our population was well poised for assessment of racial disparities in IOL. Further, a high percentage of all eligible women completed the survey, limiting selection bias. One limitation of this study was its completion at one large, urban, academic institution, possibly limiting its generalizability. Thus study was performed using a convenience sample, and thus we may not have been powered to see differences in individual survey measures. Furthermore, we are confined by the intrinsic limitations of the BSS-R, our means of determining maternal birth satisfaction in this study. No cutoff scores have been established to determine “satisfaction” using the BSS-R; thus, for the purposes of this analysis, our population’s mean score was utilized as a cut point. In addition, while both total and sub-scale BSS-R scores have been previously validated as robust tools in large, diverse populations of delivering US women, the survey is not specific to labor induction.

## Conclusions

In conclusion, this data provides several possible avenues for intervention to improve maternal birth satisfaction for IOL. First, because lower maternal satisfaction, as expected, was associated with cesarean delivery in this study, these data underscore the importance of accurate counseling regarding the possible need for cesarean section when undergoing labor induction. In addition, safe methods to reduce labor length in IOL should be explored as a means to improve maternal birth satisfaction as this study demonstrates that time to birth should be considered a patient-centered outcome. Anticipatory guidance programs could be improved for nulliparous and Black women regarding IOL, particularly to tackle the disparity seen in the “preparedness for labor” sub-scale. Finally, racial disparities in maternal birth satisfaction may contribute to racial disparities in perinatal outcomes. Further studies should explore whether interventions to eliminate disparities in maternal birth satisfaction can impact maternal and neonatal morbidity, particularly in the Black population.

## Data Availability

The datasets used and/or analysed during the current study are available from the corresponding author on reasonable request.

## Abbreviations

BSS-R: Birth Satisfaction Scale-Revised
HELLP: Hemolysis, Elevated Liver Enzymes, and Low Platelets
IOL: Induction of labor
MFMU: Maternal Fetal Medicine Units
